# Rare Disease Research Partnership (RAinDRoP): a collaborative approach to identify research priorities for rare diseases in Ireland

**DOI:** 10.12688/hrbopenres.13017.2

**Published:** 2020-11-11

**Authors:** Suja Somanadhan, Emma Nicholson, Emma Dorris, Aoife Brinkley, Avril Kennan, Eileen Treacy, Awan Atif, Sean Ennis, Vicky McGrath, Derick Mitchell, Grace O’Sullivan, Julie Power, Anne Lawlor, Paul Harkin, Sally Ann Lynch, Philip Watt, Avril Daly, Susie Donnelly, Thilo Kroll

**Affiliations:** 1 UCD School of Nursing, Midwifery and Health Systems, University College Dublin, Belfield, County Dublin, D04 V1W8, Ireland; 2 UCD School of Medicine, University College Dublin, Belfield, County Dublin, D04 V1W8, Ireland; 3 Children’s Health Ireland (CHI) Ireland, Children’s Health Ireland (CHI) at Connolly, Blanchardstown, Dublin, D01 YC67, Ireland; 4 Health Research Charities Ireland / HRCI, 12 Camden Row, Dublin, D08 R9CN, Ireland; 5 National Clinical Programme for Rare Diseases, Royal College of Physicians of Ireland, Dublin-2, Ireland; 6 National Rare Diseases Office, The Mater Misericordiae University Hospital, Dublin-7, D07 R2WY, Ireland; 7 Children’s Health Ireland (CHI) at Temple Street, Temple Street, Dublin, D01 YC67, Ireland; 8 Rare Diseases Ireland, Dublin, Ireland; 9 IPPOSI - The Irish Platform for Patient Organisations, Science and Industry, Dublin 2, Dublin, D02 XE80, Ireland; 10 NIRDP- Northern Ireland Rare Disease Partnership, 2 William Street, Northern Ireland, BT23 4AH, UK; 11 22Q11 Ireland, North Brunswick Street, Dublin, Ireland; 12 Children's Health Ireland (CHI) at Crumlin, Crumlin, Dublin, D1N5122, Ireland; 13 Rare Disease Task force, CF House, 24 Lower Rathmines Road, Dublin 6, Ireland; 14 European Organisation for Rare Diseases (EURODIS), Paris, 75014, France

**Keywords:** Rare Disease, life-course, participatory, research prioritisation, PPI

## Abstract

**Background:**

Rare diseases are individually rare, but collectively these conditions are common. Research on rare diseases are currently focused on disease-specific needs rather than a life-course perspective. The Rare Disease Research Partnership (RAinDRoP) was established in 2018 to bring together a wide variety of diverse voices in the rare disease community in Ireland and form a research partnership.

**Methods:**

A participatory multiple phase approach was used to identify research priorities for rare diseases. The research process involved three main phases: Phase I, Public Consultation Survey(PCS); Phase II, Research Prioritisation Workshop (RPW); Phase III, Public Prioritisation Ranking Survey (PRS). The time frame for the entire study was from November 2018 to June 2019.

**Results: **

In total, 240 individuals completed the phase I, of which only 96 survey participants provided information on their background,  32% (n=31) self-identified as a person living with a rare disease(s). One thousand and fifteen statements were collected, which reflected issues and shared challenges in rare diseases. MSExcel was used to gain frequencies and percentages. Phase II was focused on three main themes (1) Route to Diagnosis (2) Living with Rare Disease (3) Integrated and Palliative Care. 42 participants engaged at each workshop. Seventy-five individuals completed the phase III prioritisation ranking survey and ranked the top 15 research priorities.  The top five priorities were (1)Support at the time of diagnosis, (2) Diagnostic test for rare diseases (3)Education and training (4) Patient voice (5) Data sharing and integration of services for rare diseases.

**Conclusions:**

The research priorities identified here for rare diseases were developed jointly in collaboration with patients, families, healthcare professionals and policymakers. So, we encourage researchers, funding bodies and other stakeholders to use this priority list as a guiding document for future research work to improve the health and lives of people living with rare diseases.

## Abbreviations

Rare Disease Research Partnership (RAinDRoP); Health Research Charites In Ireland (HRCI); Health Research Board Ireland (HRB); Patient and Public Involvement (PPI); University College Dublin (UCD); European Reference Networks (ERNs); European Joint Programme (EJP); International Rare Diseases Research Consortium (IRDiRC); General Data Protection Regulation (GDPR)

## Introduction

Rare diseases are individually unique, but collectively they share substantial unmet health and social care needs ^1, 2^. These pose a significant public health challenge and many of these conditions have genetic causes ^1, 2^. Definitions vary, with some definitions depending solely on the number of people living with certain diseases ^1^. In Europe, they are defined as conditions that affect fewer than five people in 10,000 ^1^. Individually, these numbers might appear small. However, collectively, these conditions affect an estimated 30 million Europeans and 20 million Americans and create significant challenges for affected individuals and their families, health and social care systems and society as a whole ^3– 5^. To date, approximately 7,000 rare diseases have been identified, with estimates of around 300 million people affected worldwide. An estimated 95% of rare diseases have no approved treatment ^6, 7^. Since 2001, only 140 orphan medicines have been used in the European Union for treatment. Of these 60% were designated for use in paediatric populations ^7– 9^. Rare diseases are challenging for clinicians in terms of reaching a conclusive diagnosis and determining an appropriate course of treatment due to their low prevalence, heterogenicity and complex nature ^10, 11^. Considering these challenges, the European Commission (2017) has established the first European Reference Networks (ERNs) across Europe, which share knowledge and resources concerning diagnosis, treatment and support ^12^. The European Commission also supports research, development and innovation in this area through projects funds and joint actions ^13, 14^. Currently, 24 ERNs are working on a range of thematic issues involving highly specialized complex care, aiming to facilitate access to diagnosis, treatment and provision of affordable, high-quality and cost-effective healthcare ^12^.

Research on rare diseases is a top priority by the European Commission, and to date more than 1.4 billion euro has been invested in 200 or more research and innovation projects ^1^. However, at the national level in Europe, rare diseases are currently under-researched and under-resourced, and no uniform standards are governing the collection, management or use of rare disease data registries ^1, 5, 15^. As specialist expertise is scarce, patients and their families may find it challenging to gain access to diagnostic testing and treatments. Psycho-social support is also limited ^10, 16^, leaving families feeling isolated and vulnerable ^4, 5^. The research into rare diseases and holistic care for people living with rare diseases are now an EU Commission priority ^1^. In Ireland, the National Rare Disease Plan ^2^ contains the recommendation to develop a rare disease research network in line with its strategic priorities. It emphasizes that “ *the needs and experiences of people with a rare disease are recognized, understood and addressed within all aspects of the Irish health system, including policy, services and research/information system*” ^2^, p.8. In 2011, the European Commission jointly with the US National Institutes of Health (NIH) launched the International Rare Diseases Research Consortium (IRDiRC) ^16^. The Consortium strives to strengthen international collaboration in the area of rare disease research. Specifically, the IRDiRC's vision for the period 2017–2027 is to ensure that all people with rare diseases receive a timely diagnosis, as well as appropriate care and treatment within the first year of diagnosis. 

The Health Research Charites In Ireland (HRCI), formerly known as Medical Research Charities Group (MCRG), brings many charities together and supports collaborative health research. The HRCI and the Health Research Board Ireland (HRB) have been operating a joint funding scheme since 2006, and as of 2018, they have funded 125 projects ^17^. Cody(2018) ^17^, p.5 highlighted in a recent workshop on clinical research in rare diseases by HSE clinical strategy and programmes division that nearly two-thirds of HRB-funded rare disease research projects are focused on applied biomedical research or clinical research projects in rare diseases. Given the low prevalence and considerable heterogeneity of rare diseases, it can be challenging to focus research on specific conditions and thus, identifying shared research priorities across rare diseases can increase the impact of research in this area. It is, therefore, imperative to identify top research priorities for rare diseases which could gain consensus about areas focused on a life-course perspective rather than a disease-specific need.

There has been a lack of discussion on the research topics that should be prioritised and gaining consensus about research priority areas is timely and important. Health research prioritisation is a critical element of health system strengthening efforts to maximize impactful research and ultimately, better care quality and health outcomes ^18– 20^. In alignment with the *National Rare Disease Plan*, a Rare Disease Research Partnership (RAinDRoP) was formally established in 2018. RAinDRoP is a collaborative research partnership of the rare disease community in Ireland, and it comprises of academic researchers, health professionals, rare disease advocates and families living with rare diseases. The patients and their families are often the experts in rare diseases, due to the nature of the conditions and lack of expertise. Hence, the greater importance of including the patient and carer’s voice in the priority setting exercise, rather than just academics and HCPs. This patient and public involvement (PPI) research partnership placed the lived experience of people with rare diseases at the centre as opposed to a biomedical or condition-specific orientation. As the recognition for the PPI in Irish health and social care research grows, we want to make sure that the patient voice is central rather than merely the professional or academic view and expertise. The identification of shared research priorities will strengthen the health system overall as this approach will likely translate into better immediate benefits for patients ^18– 20^. Biomedical research is critical for rare diseases, but the impact can take many years to reach patients and so this type of work can help families in the interim. This article reports on a rare disease research prioritization exercise. The initiative was led by the University College Dublin (UCD) in Ireland and supported by HRB Ireland, the National Clinical Programme for Rare Diseases, Rare Diseases Ireland, HRCI and The Irish Platform for Patient Organizations, Science and Industry (IPPOSI).

The aim of the RAinDRoP initiative was two-fold. First, RAinDRoP was established as a collaborative research partnership and evolving network in response to the *National Rare Disease Plan* for Ireland to ensure relevantly, focused and coherent research informed by the needs and experiences of people living with rare diseases. Second, to identify rare disease research priorities for Ireland from multiple stakeholder perspectives.

### Ethical considerations

This study received an exemption from full ethical review by the Office of Research Ethics at UCD. The Ethics Exemption Reference Number (REERN): LS-E-19-32-Somanadhan.

## Methods

Having considered the various methodologies and schools of thoughts, participatory multiple method was chosen as a suitable methodological approach for this project. We felt this approach would be the most appropriate to reflect and promote participation from the patients and public involvement (PPI) perspective to focus and identify research priorities that address uncertainties of living with rare diseases. Participation in this study means that individuals are involved in Rare Disease research Partnership (RAinDRoP) was engaged in a meaningful way from the beginning of the process with a focus to improve the quality of the patient-focused rare diseases research and its impact. An expert group was formed to oversee this research prioritisation exercise and this group composed of members of the rare disease taskforce, patient organisation representatives (n=3); patients and families living with rare diseases (n=3); members of the National Rare Disease Office in Ireland (n=2), academics (n=2), researchers (n=2), healthcare professionals (n=2).

The patient and family voice have been integral to this work from the start and adopted the priority setting partnership process to conduct multiple rounds of stakeholder recruitment, engagement and research prioritization ^21^. With that in mind, equal representation from patients, carers, health and social care professionals, academics, representatives for rare disease support organizations/non-governmental organizations, government agencies and policymakers were invited to join initial discussions.

**Figure 1.**
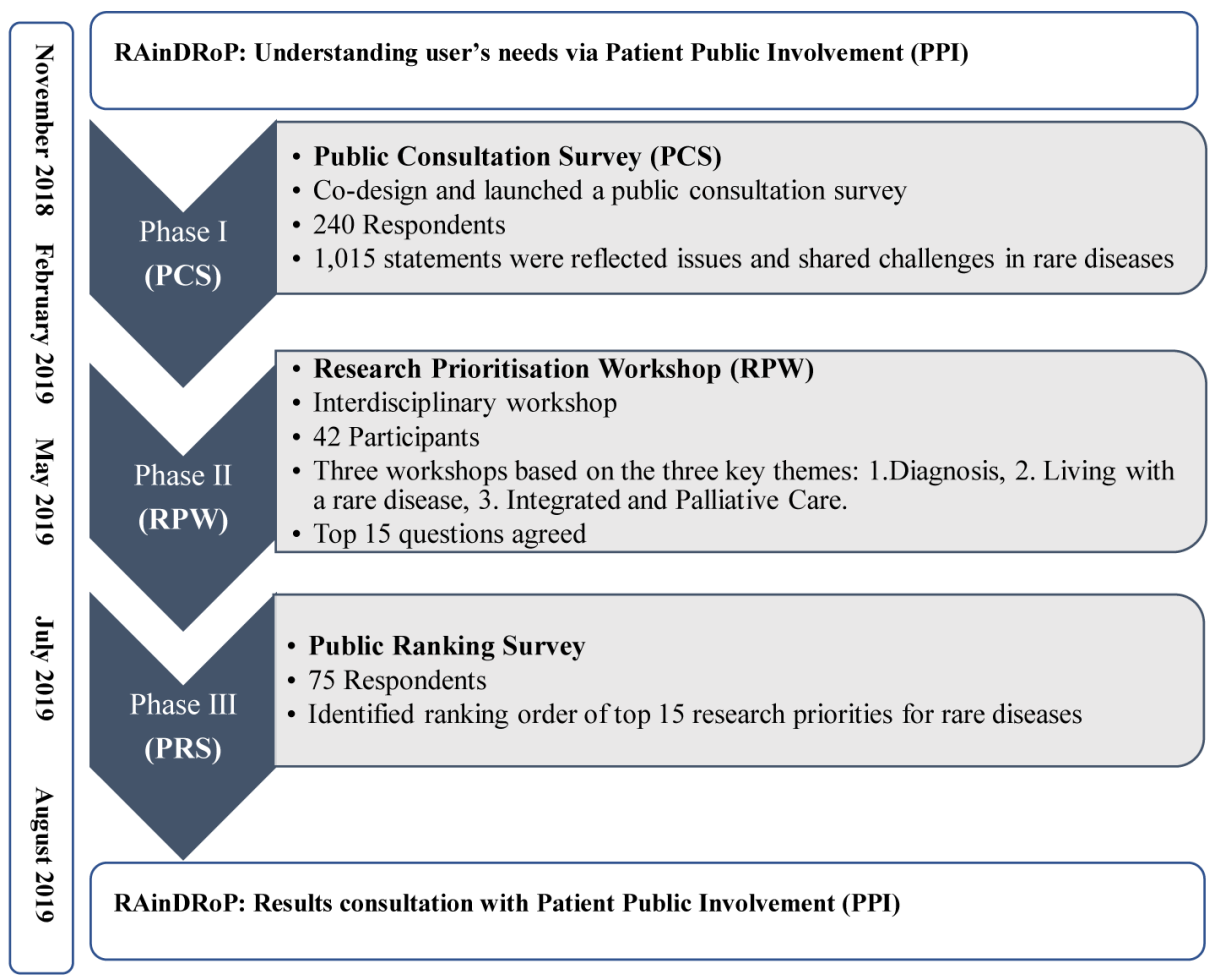
Three phases of the priority setting exercise scheduled participatory approach.

The research process involved three main phases (1) Public Consultation Survey (PCS), (2) Research Prioritisation Workshop (RPW), (3) Prioritisation Ranking Survey (PRS). Three phases of the priority setting exercise are listed in Figure 1. 

### Phase I: Public consultation survey (PCS) on Research in Rare Diseases in Ireland

The process of research priority setting for rare diseases can be complex due to heterogeneity, and each rare disease impacts a small population. However, collectively many individuals are affected by these conditions. Therefore, identifying research prioritisation on each rare illness can be challenging in a single priority setting to identify, address and integrate the different perspectives from estimated 7000 rare diseases. Hence, the expert group collaboratively designed the initial framework of the survey from a life-course perspective rather than a disease-specific focus. The focus was on *“What questions would you like to see answered by Rare Disease research?”*. The expert group identified and co-designed list of priority areas for the survey through a review of existing literature and policies relevant to rare disease. Six key topics were chosen, and these are listed in Table 1.

**Table 1.** Survey thematic priority areas and related questions.

	Priority areas
**1.**	**Diagnosis** What question(s) about dealing with the diagnosis of Rare Disease would you like to see answered by research?
**2.**	**Day-to-day life** What question(s) about managing day-to-day life with Rare Disease would you like to see answered by research?
**3.**	**Treatment** What question(s) about the treatment of Rare Diseases would you like to see answered by research?
**4.**	**Self-management / overall management** What question(s) about the self-management/overall management of Rare Disease would you like to see answered by research?
**5.**	**Integrated / holistic care** What question(s) about the integrated care/holistic care of Rare Disease would you like to see answered by research?
**6.**	**Palliative care** What question(s) about the palliative care service for advanced Rare Disease would you like to see answered by research?

The survey was officially launched on the National Rare Diseases Day on the 28 ^th^ of February 2019. A paper-based and online version using SurveyMonkey® was made available for four weeks (February to March 2019). Social media (Twitter, LinkedIn, Facebook) was utilised to share participant information leaflets ( *Extended data*: File 1 ^22^) and the online survey ( *Extended data:* File 2 ^22^). Content analysis ^23^ was used to identify the main themes that emerged from the survey respondents.

The survey asked respondents to think of questions they would like to see answered by rare disease research in relation to the six topics identified by the expert group. There was also an open field to capture any other questions respondents felt were important. The expert group met to examine the research issues and statements identified in the survey. Each expert group individually scored statements specific to each theme (Diagnosis, Day to Day Life, Treatment, Self-Management, Integrated and Palliative care, and other). MSExcel was used to gain frequencies and percentages, and any statements that had received a total score of above 50% were included in 10–12 researchable questions or statements per the theme. This was to reduce the number of questions/statements to a manageable level. From this ranking, a total of 29 themes or statements identified from the surveys were brought forward for discussion at the phase II workshop.

As the data was collected anonymously, the UCD Human Research Ethics Committee approved an ethics exemption for the conduct of the phased priority setting exercise. Participants did not receive any incentives for completing the survey. Participants indicated written consent to participate at the beginning of the survey.

### Phase II: Research Prioritisation Workshop (RPW)

The phase II RPW took place at UCD. Prior to the event, a short animation ^24^ was produced to promote the event and shared on social media to raise awareness. Targeted invitations to attend the workshop were circulated by the Rare Disease Taskforce, Rare Disease Ireland, National Clinical Programme for Rare Diseases, and IPPOSI. There was a focus on creating a cross-section of individuals from service providers, service users, and the public perspective. Participants included those living with rare diseases, family, carers, clinicians, genetics/scientist, policymakers, research funding bodies, interdisciplinary healthcare and social care professionals, and researchers with a particular interest in rare diseases. Eligibility criteria were as follows: English speaking; 18 years and older; and able to provide informed consent to participate. There was a clear focus in this workshop to achieve gender balance, leading to a 50:50 split of men and women. It was also ensured that minority ethnic groups were included during the invitation.

The workshop sessions were chosen with a life course perspective in mind. The focus of these sessions predominantly centred around three themes distilled by the expert group from phase I (see below). On the morning of the workshop, each theme was introduced by expert speakers, so that participants had an opportunity to learn more about the three themes, ask questions and share knowledge and experiences ( *Extended data*: File 3 contains the RPW agenda ^22^). The three thematic sessions based on the results of phase I are as follows:

*Theme 1: Route to Diagnosis:* This session focused on research questions about obtaining a timely diagnosis, methods of diagnosis, as a basis for bespoke treatment options. Aside from basic genetic research challenges, the session also focused on how to communicate diagnosis and treatment options to patients and their families. 

*Theme 2: Living with and Caring for Rare Diseases (Experience/Quality of Life/Psycho-social needs):* This session examined the patient experience of living with a rare disease journey rather than a disease-specific experience. 

*Theme 3: Integrated and Palliative Care: Providing integrated care pathways*. The session aimed to identify integrated care challenges about rare diseases and areas for research.

**Figure 2.**
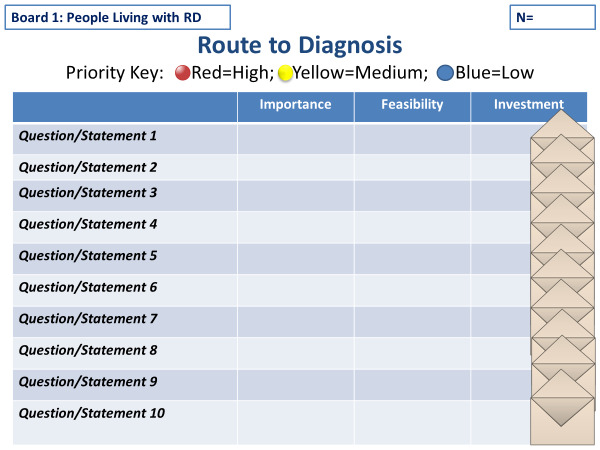
Example of a prioritisation board used in phase II.

The afternoon of the workshop focused on creative conversations in smaller interdisciplinary and heterogeneous groups. In-depth discussions following the prioritisation exercise were referred to as ‘RAinDRoP cafés’. Two ‘café hosts’ per session guided the groups through the process. Each group had approximately 40 minutes to discuss a theme (either Route to Diagnosis; Living with Rare Disease; or Integrated and Palliative Care). Café agenda was as follows:

- Café hosts introduced the session theme and gave participants a pack that consisted of handouts of each theme and examples, sticker sets (blue/low importance, yellow/medium importance, red/high importance; 10 of each sticker colour per person) and play money (one set per person consisting of: 1 x €50 2 x €20, 1 x €10, 1 x €5).
- Aspects that contribute to feasibility and whether they would impact the prioritization of the theme were discussed, e.g. cost, availability of resources, capacity to build resources, electronic health records, samples sizes, expertise, local knowledge.
  A group discussion was then performed concerning what attributes they attribute importance to for research in the given theme.
- Finally, participants were explicitly asked to rate questions/statements (10–12 per theme) identified through the PCS in phase I in terms of their importance and feasibility. Participants were also asked how much they would invest in these questions. The colour-coded stickers were used to indicate the degree of importance and feasibility, and the money was used to 'cash invest' into questions/statements displayed on large poster boards (see Figure 2).

Three prioritisation poster boards per session were available to determine similarities and differences of ratings between the three groups:

*Board 1:* People living with a rare disease (PwRDs) (including family members, carers, patient advocates, advocacy groups).

*Board 2:* Health Care Practitioners (HCPs), including all clinical policymakers.

*Board 3:* Academic, including researchers, academic policymakers, research managers

Participants were then given colour-coded stickers to assign a level of importance, and feasibility to each subtheme. To further clarify their decision-making and encourage active participation, each participant was given play money and asked to invest it as they saw fit – to put their money where their mouth is! However, we only considered their score for importance when generating the top priorities due to indifference approach across three workshops. 

There was a total of 28 research themes or statements identified from the at the phase II prioritisation workshop. We identified top 10 research priorities that can inform the direction of rare disease research over the next seven years. However, we noticed from the percentage scale from the respondents from the three workshop (n=42) top priorities list were scored almost identical. The expert group agreed to create a list of 15 priorities instead of 10 and that should also be sent out in a public validation survey.

### Phase III: Prioritisation Ranking Survey (PRS)

The top 15 research priorities defined during phase II were opened to the broader public for ranking by priority. There was no formal target sample size set for this survey. The ranking survey was also constructed with SurveyMonkey ® ( *Extended data:* File 4 ^22^). The PRS link was distributed by email and the survey was also available in paper format if participants preferred. The RAinDRoP expert group members and partners were asked to promote the survey to stakeholders via email, relevant meetings, social media, web sites, and any other opportunities that arose. A social media promotion plan was developed, similar to phase I, and there were no incentives offered for return of the survey. Respondents were asked to rank the top 15 research priority areas in order of importance. All respondents’ votes were considered equally valuable, and no weighting system was applied. Based on respondent rankings, we identified which of the top 15 rare disease research priorities were the most important. The survey was live for four weeks between May 2019 to June 2019.

## Results

Each phase generated findings that informed the subsequent phase. Project timelines are contained in *Extended data: *File 5 ^22^.

### Phase I: PCS

**Figure 3.**
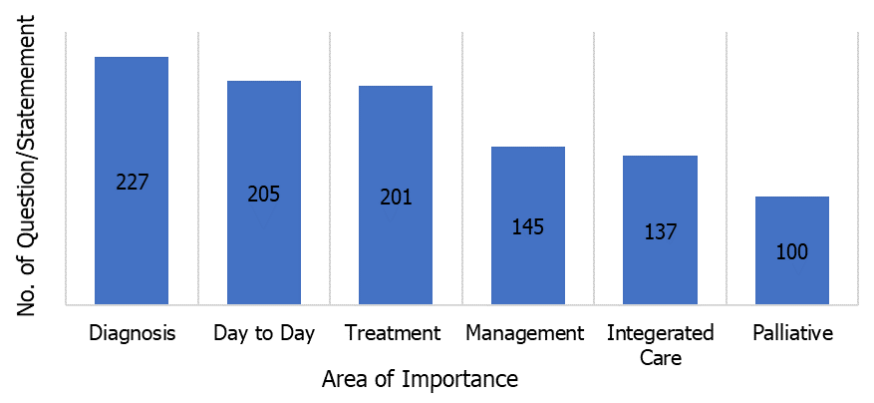
Survey responses (n=1015) identifying shared challenges in rare diseases by topic.

In total, there were 240 respondents to the survey. However, a total of 144 survey participants skipped their answers to describe their category. In total, 96 survey participants provided information on their background: 32% (n=31) self-identified as a person living with a rare disease(s); 32% (n=31) self-identified as health and social care professionals (e.g. doctors, nurses, consultants, researchers, managers); 19% (n=18) self-identified as a friend or family member of a person living with a rare disease; 11% (n=10) self-identified as carers of a person living with a rare disease; and 6% (n=5) indicated 'other' (including academic researchers). A total of 1015 statements were submitted through the survey, which reflected issues and shared challenges in rare diseases ( *Underlying data:* File 1 ^22^; Figure 3). Most research questions proposed by participants were related to ‘diagnosis’, e.g. “What is the best way to tell someone about the diagnosis?”; followed by ‘day-to-day life’ with rare disease, e.g. “How do rare diseases affect family life?”, and ‘treatment’, e.g. “How often do GPs or consultants put patients with a rare disease forward for clinical trials?". Initial grouping of questions into themes by the expert group are available in *Underlying data:* File 2 ^22^.

### Phase II: RPW

Sixty-two (n=62) people participated in the event. These included living with rare diseases (n=15), family (n=10), carers (n=10), clinicians (n=10), genetics/scientist (n=5), policymakers (n=5), research funding bodies (n=2), interdisciplinary healthcare and social care professionals (n=15), and researchers with a special interest in rare diseases (n=10). Of the 62 participants, 42 took part in the RAinDRoP café portion of the day. We assigned them to three cafés (see Table 2). Each group contained a cross-section of health care professionals (HCPs), people living with a rare disease (PwRD), including family members, carers, patient advocates and advocacy groups, and others, including academics, researchers, academic policymakers and research managers. 

**Table 2.** Composition of the three cafés in phase II.

Café 1. Route to Diagnosis	Café 2. Living with Rare Diseases	Café 3. Integrated and Palliative Care
Healthcare practitioners (n=4) People living with a rare disease (n=5) Others (n=5)	Healthcare practitioners (n=5). People living with a rare disease (n=5) Others (n=5)	Healthcare practitioners (n=5). People living with a rare disease (n=4) Others (n=4)

Each participant was given sticker sets and play money (as described in the Methods). Participants were then asked to assign a level of importance and feasibility to each subtheme using the colour coded stickers. To further clarify their decision-making, each participant was asked to invest the play money as they saw fit – to put their money where their mouth is! Data was sorted by % of high priorities, and then % of euro investment. Applied heat mapping to show which themes are more dominant within the high priority group are available as *Underlying data*: File3 ^22^.

The RPW identified the top subthemes from each café within each main theme by importance. The top priority refers to the number (count) of “high priority” stickers. Each Cafe distributed stickers in a variety of three colours (red, yellow and blue). In Cafes 2 & 3 red stickers were used to denote “high” priority and in Café 1 blue stickers were used to denote “high priority”- the colours are nominal, we refer to them as high, medium or low priority stickers.

Café 3 (Integrated and Palliative Care) had two subthemes that were equally ranked in 1 ^st^, 2 ^nd^ and 7 ^th^, 8 ^th^ position. ‘ *Data sharing and integration*’ and ‘ *co-designing services*’ ranked evenly as high importance (24 high importance stickers each) but euro investment was €945 for data sharing and €365 for co-designing services ( Table 3).

**Table 3.** Research Prioritisation Workshop café top 10 priorities.

Café: Top Priorities (i.e. based on top three subthemes within each cafe)	Importance assigned to each theme		Euro	
	n	%	n	%
Data sharing and integration (Integrated and Palliative Care)	24	21	945	44
Co-designing services (Integrated and Palliative Care)	24	21	365	17
Psychosocial impact (Living with Rare Disease)	19	18	590	22
Support at the time of diagnosis (Route to Diagnosis)	19	16	170	6
Transition services (Living with Rare Disease)	16	15	425	16
Economic impact (Living with Rare Disease)	16	15	245	9
Community-based services, treatment, multi-morbidity (Integrated and Palliative Care)	15	13	230	11
Evidence-based models of integrated care (Integrated and Palliative Care)	15	13	180	8
Role of infrastructure in diagnosis (Route to Diagnosis)	15	13	525	18
Psychosocial impact of a diagnosis (Route to Diagnosis)	14	12	570	20

The RPW also revealed differences in prioritisation between Health Care Professionals (HCPs), People living with Rare Diseases (PwRDs) and others. The two priorities equally identified by these three groups were ‘ *co-designing services’* and ‘ *data sharing and integration’*. PwRDs scored high importance score (9), and HCPs scored (7) for the theme ‘ *support at the time of diagnosis*’ compared to others, and they scored (3). PwRDs identified the ‘ *best way to deliver diagnosis’* as their top research priority. They scored it 10 while respondents of the ‘others’ category assigned a score of 0 and HCPs gave a rating of 3. ‘ *Patient voice*’ as part of research was highly prioritised by the PwRD (9), and it is worth noticing that this was HCPs least prioritised theme with a score = 1. The different views expressed in the scoring illustrate the importance to including all stakeholders in the research prioritisation process. Table 4 highlighted overall scoring from the RPW and *Underlying data*: File 4 ^22^ highlights priority ratings in terms of importance by all café groups in phase II.

**Table 4.** Phase II: Research Prioritisation Workshop (RPW).

	RPW task: Participants asked to assign “high-priority” stickers to indicate the importance of the following areas for research		
	Top priorities from Research Prioritisation Workshop (RPW)	(n) stickers	%
1	Data sharing and integration	24	7%
2	Co-designing services	24	7%
3	Support at the time of diagnosis	19	6%
4	Psychosocial impact	19	6%
5	Transition services	16	5%
6	Economic impact	16	5%
7	Role of infrastructure in diagnosis	15	4%
8	Community-based services, treatment, multi-morbidity	15	4%
9	Evidence-based models of integrated care	15	4%
10	Psychosocial impact of a diagnosis	14	4%
11	Best way to deliver a diagnosis	13	4%
12	Family experience	13	4%
13	Palliative Care at-home	13	4%
14	Diagnostic tests	12	4%
15	Patient Voice	12	4%
16	Education and training	11	3%
17	Timeframes to diagnosis	11	3%
18	Psychosocial services	11	3%
19	Understanding incidence and prevalence	9	3%
20	Role of diagnosis in understanding the impact of disease	9	3%
21	Education and training	9	3%
22	Equitable and timely access, provision of palliative care	8	2%
23	Screening and risk	7	2%
24	Palliative Care education and training of HCPs	7	2%
25	Preparation for bereavement, acceptability of the palliative care role in end-of-life	7	2%
26	Information and awareness	6	2%
27	Information for families and patients	5	1%
28	Advanced care planning	1	0%
		341	100%

### Phase III: Prioritisation Ranking Survey (PRS)

Following the Priority Setting workshop, the team decided to extend the ranking of priorities to the wider public using an online survey. All topics from the research prioritisation workshop were included, and respondents were asked to rank these areas in order of importance. From this, a final set of research priorities were identified to inform the future direction of rare disease research. There were 75 total responses to the PRS. However, 27 survey participants did not complete the demographic section. A total of 48 survey participants described their categories: 67% were from the Leinster province; 30% (n=14) self-reported as a friend or family member of someone with a rare disease, whereas 19% (n=9) self-reported as a PwRDs. *Underlying data*: File 5 ^22^ provides priorities ranked in the first position by respondents during the PRS. Of the 15 topics for ranking, ‘ *support at the time of diagnosis*’ ranked the highest with 23% (n=10) of respondents identifying this as a top priority area. ‘ *Diagnostic testing for rare disease*’ and ‘ *education and training* *’* also ranked highly at 14% (n=6) each. Research into ‘ *how best to deliver a rare disease diagnosis* *’* was not identified as a priority by any of the survey respondents. It may be the case that respondents felt this was already captured by the theme ‘ *support at the time of diagnosi* *s*’. Table 5 highlighted Top 15 Rare Disease research related themes from phase III PRS. Table 6 contains the top 15 rare disease research related priority themes in full.

**Table 5.** Phase III: Public Ranking Survey (PRS).

Q: Which research question or area would you like to see prioritised for Rare Diseases? Use the drop down to rank in order of your preference.		
Top 15 Rare Disease research related themes from PRS	(n) respondents	%
Support at the time of a Rare Disease diagnosis	10	23%
Diagnostic tests for Rare Diseases (e.g. Use of genetics, Stratified medicine/ molecular targeted therapies, gene therapy etc.)	6	14%
Education and Training (e.g. health and social care professionals, school, GP and patient information and understanding of their illness and management)	6	14%
Patient Voice (eg: How to include the child’s voice in relation to their care)	4	9%
Data sharing and integration of services for Rare Diseases	4	9%
Economic impact of living with Rare Diseases (e.g. healthcare costs, transportation costs, education costs, loss of earnings, etc.)	3	7%
Psychosocial impact of living with Rare Diseases (e.g. physical functioning, psychological, social and mental health and quality of life etc.)	2	5%
Community based services and treatment for Rare Diseases	2	5%
Evidence-based models of integrated care for Rare Diseases	2	5%
Family experience of living with Rare Diereses (e.g Parents, mother, father, siblings and grandparents experience of living and caring and life-course transitions)	1	2%
Transition services for Rare Diseases (e.g barriers and enablers for transitioning from paediatric to adults’ services)	1	2%
Co-design of (research, services, information, dissemination) for Rare Diseases	1	2%
Psycho-social impact of a Rare Disease diagnosis	1	2%
Role of infrastructure in diagnosing a Rare Disease (e.g Registry/ERN Centres of excellence)	1	2%
Best way to deliver a Rare Disease diagnosis (e.g. mail. phone, in person (consultant, GP, Nurse, other)	0	0%
	44	100%

**Table 6.** Top 15 rare disease research priorities for rare diseases in Ireland.

1	Support at the time of a rare disease diagnosis	Support at the time of diagnosis was a top priority in the public ranking survey. At the workshop, participants discussed the importance of communication at the time of diagnosis and issues, such as ‘who is the most appropriate person to deliver a rare disease diagnosis?’ and ‘how should it be delivered?’. Participants also considered that not having a diagnosis created a blockage to care and felt that more research was needed to explore the impact of this.
2	Diagnostic tests for rare diseases (e.g. use of genetics, stratified medicine/molecularly targeted therapies, gene therapy etc.)	Diagnostic testing was ranked as the second most important research area by survey respondents. The category encompasses the development of diagnostic genetic procedures for stratified medicine, targeted molecular therapies and gene therapies. EURORDISCARE 2 (2007) ^25^ showed that 25% of patients with one of eight rare diseases had to wait between 5 and 30 years for a confirmed diagnosis. During that time, 40% received an incorrect diagnosis. Accurate and timely diagnosis is essential.
3	Education and training (e.g. health and social care professionals, school, GP and patient information and understanding of their illness and management)	Understanding and improving the education and training of people and institutions who interact with the rare disease community is a priority. Included were health and social care professionals who treat and manage people with rare diseases and the relevant institutions (i.e. schools and workplaces) that also need to understand their illness. Further, this priority also included the education needs of people living with a rare disease in this category
4	Patient voice (e.g. how to include the child's voice about their care)	The inclusion of the patient voice is an essential element in the development of rare disease research priorities. The rare disease research community must continue to focus on developing research grounded in first-hand experiences and insights of patients, using patient and public involvement methods.
5	Data sharing and integration of services for rare diseases	Data sharing and integration was a top priority for rare disease research. In the workshop, it received the highest importance ratings and attracted the most substantial cash investment. During the café discussions, participants talked about a lack of infrastructure to share data, and the implications of General Data Protection Regulation (GDPR) on data sharing across disciplines and sites and in terms of learning and linking in with other partners, in other countries to create high-quality research.
6	The economic impact of living with rare diseases (e.g. healthcare costs, transportation costs, education costs, loss of earnings, etc.)	Participants would like to see more research into the economic impact of living with a rare disease. Indirect cost measures should be part of this effort (e.g. excess family expenditure for transportation, home adaptations, etc.).
7	Psycho-social impact of living with rare diseases (e.g. physical functioning, psychological, social and mental health and quality of life etc.)	The psycho-social impact of living with a rare disease was another top research priority. This encompasses the effects on education and employment opportunities, stigmatisation, friendships etc.
8	Community-based services and treatment for rare diseases (Integrated and Palliative Care)	Community-based services, treatment, multi-morbidity was discussed in terms of delivering care closer to home in an integrated way. This category included palliative care needs for individuals living with rare progressive and complex illness.
9	Evidence-based models of integrated care for rare diseases	Evidence-based models of integrated care were discussed in the workshop as part of integrated and palliative care. Participants suggested that the rare disease research partnership should explore what evidence for pathway and integrated care models for rare diseases and other conditions may have been developed in other countries and learn from these experiences.
10	Family experience of living with rare diseases (e.g. parents, mother, father, siblings and grandparents experience of living and caring and life-course transitions)	The impact of rare diseases on family members other than the patient is currently under-researched. A Europe-wide survey on juggling care and daily life with a rare disease, conducted by EURORDIS-Rare Diseases Europe via its Rare Barometer Voices platform (May 2017) ^26^, identified that seven in ten patients and carers reduced or stopped professional activity due to their or their family member's rare disease, and this group are three times more likely to report to be unhappy or depressed than the general population.
11	Transition services for rare diseases (e.g. barriers and enablers for transitioning from paediatric to adults' services)	The transition of services was discussed not only in terms of transition of care but also the shift of responsibility from the parent to the child, or young adult. Potential areas of research included the cost of poorly managed transition and the transfer of information from paediatric to adult services and associated challenges presented by GDPR.
12	Co-design of research, services, information, dissemination for rare diseases	Participants regarded research into and involving collaborative service design as a priority. This approach enables academics, health and social care professionals and patients and carers to co-design services and care pathways.
13	Psycho-social impact of a rare disease diagnosis	Research into the psycho-social impact of a rare disease. The diagnosis was a high priority for participants, especially for those living with a rare disease. Participants expressed that this is a vulnerable point in the lives of people living with a rare disease ^4^ and that better understanding of what is required to support them through this period would be valuable.
14	Role of infrastructure in diagnosing a rare disease (e.g. Registry/European Reference Networks Centres of Excellence)	Role of infrastructure in diagnosis was a high priority. This referred to the role of European Reference Networks (virtual networks involving healthcare providers across Europe), and patient registries. Health care practitioners were particularly concerned about the feasibility of developing infrastructure around diagnosis.
15	Best way to deliver a rare disease diagnosis (e.g. mail, phone, in person (Consultant, GP, Nurse, other))	The best way to deliver the diagnosis was an issue that was consistently highlighted throughout this process. Notably, the need to improve communication skills among health care professionals was one of the top education and research priorities.

## Discussion

Following established a national rare disease research partnership (RAinDRoP), we identified research priorities for rare diseases through PPI, which aimed at improving the health and wellbeing of people living with rare diseases across life-span. This was achieved with close and continuous engagement with the person living with rare diseases, families, carer, healthcare professionals to ensure that research priority relevance for improving quality and long-term management for any form of rare diseases journey. These priority topics were developed from input solicited through the multiphase process such as public survey, research prioritisation workshop and prioritisation ranking survey. The 15 rare disease research priorities addressed aspects of rare disease diagnostic challenges, integrated care and holistic care services, data integration and data sharing focused on European Reference Network, patient voice and patient focused research and service approaches, education and training needs for healthcare professionals and community, psychosocial and economic impact of living with rare diseases, transition services for both school and healthcare transition, family experiences of caring for people living with rare diseases, co-designing services using PPI input and the role of infrastructure in diagnosing a rare disease. We agreed to keep priorities are categorised within broader themes to represent the view of the patient, family and healthcare professionals across life-span, rather than narrow it down to a research question specific priority with a focus on a particular rare disease. We felt these research priorities represent key strategic areas that are executional in nature despite of settings.

The top 5 research priorities are discussed as follows: (1) Support at the time of diagnosis was ranked as a top research priority following a public ranking survey. This finding is consistent with recent studies ^4, 27, 28^, as they reported the need to prioritise and address the inadequacy of communication skills among healthcare professionals, especially during the initial diagnostic disclosure. Families reported feelings of frustration and their concern about professionals’ lack of understanding of the specific rare disease could have a negative impact on their trust in the health care system. (2) Diagnostic test for rare diseases was the second research priority list. The diagnostic odyssey for individual living with a rare disease has been reported ^23, 24, 28^, and it can reported as a confusing and chaotic experience. There are a number of initiatives have been created to support the undiagnosed rare disease community, for example: Undiagnosed Disease Network International (UDNI), SWAN Europe ^23, 24^. EURORDISCARE 2 (2007) ^25^ showed that 25% of patients with one of eight rare diseases had to wait between 5 and 30 years for a confirmed diagnosis. During that time, 40% received an incorrect diagnosis. Accurate and timely diagnosis is essential for early recognition of rare genetic disease and management preventive life- long impairments, which means the newborn screening programme to be uniformly applied to across the European member states to offer equal quality of care and service for every child born in Europe ^25, 26^. (3) Education and training were identified as the third research priority, and this is consistent with recent studies ^28, 29^ and highlighted the need for providing accessible education and training for families and the community. Those studies have reported the requisite for primary care physician’s knowledge and understanding of rare diseases and the need to create information sessions for professionals and students. (4) The patient voice was the fourth top priority needs for research in the field of rare diseases. This was focused very much to encourage the participatory engagement of individual living with rare diseases, and that will enhance understanding of their day to day life experiences in the service decision-making process. There is growing recognition of the value of collecting and sharing data on a globally. (5) Data sharing and integration of services for rare diseases were ranked as the top 5 ^th^ research priority.

This priority exercise was a co-designed at every stage of the process from the concept design, survey design, thematic analysis, workshop and the final ranking. The exercise aimed to maximise the impact for the rare disease community in Ireland, reduce duplication of effort and promote collaboration and partnership between clinicians, patients and their families and researchers ^30^. Therefore, the public ranking survey was important to offer an equal opportunity to respond to this consultation process. Hence the change in position of priority list doesn’t affect the overall process. Identifying research priorities for rare diseases at a national level can have the most significant impact on national rare disease policy ^2^, and its implementation and evaluation are critically necessary to foster research and development in the field of rare diseases. Research is one of the major pillars of a national plan on rare disease ^2^. The prioritisation workshop created an opportunity for information-sharing and open dialogue around the challenges faced by a rare disease, as well as its future direction. Relationships built between researchers and those with lived experience have the potential to extend to future collaborations. It mobilises information and expertise sharing and can help sustain these efforts through collaborative networking funding schemes such as the European COST ACTION, E-RARE, European Joint Programme (EJP) ^14, 15, 31^.

Europe-wide priorities for rare diseases have been identified by E-rare and EJP ^14, 15, 31^. EJP identified the need for better epidemiological data and information on the natural history of rare diseases ^14^. Most of the survey participants were basic researchers and clinical scientists (85%) in contrast to survey participants in our prioritisation exercise. The RAinDRoP research prioritisation offered an ongoing process of participation, involvement and engagement across various members including clinicians, patients, families, academics, researchers and NGOs. These process of participation, involvement and engagement are accurately managed and applied correctly at the RAinDRoP research activity using the participatory approaches, by asking the question, *‘Who should be involved, why and how?’* for each phase of this process an appropriate and context-specific participatory approach was developed ^32^, p.1.

The research prioritisation activity enhanced relationships between researchers, public and health care professionals, thereby increased public knowledge and awareness, understanding and support of rare disease research. This prioritisation process stimulated the development of a rare diseases research consensus group, which included national and international experts from the clinical, academic, professional disciplines and patients and caregivers. The utilisation of modified priority setting partnership methodology raises the benchmark for quality and good practice for research priority developing partnership. The PPI ultimately increased accountability and transparency of research design, collaboration and knowledge translation through participation, involvement and engagement.

### Limitations

The research priority setting exercise itself has cleared several key limitations. It is important to note that the number of research priorities identified for rare disease is not just focused on a specific disease category. However, the priorities are broadly related to shared challenges of Rare Diseases from a life-course perspective.

Participants across all three phases are not necessarily representative for all stakeholder groups nor for the entire rare disease community in Ireland. Many individuals and families living with rare diseases may not have been able to participate in this exercise. Similarly, from the health and social care field, advocates or academic experts may have been missed. Despite various endeavours to make the workshop itself as accessible and inclusive as possible, it may still have excluded individuals who could not attend on the day.

In the prioritisation process, the focus on research may have been lost for some participants. This became evident in some of the survey responses and at the workshop discussions where distinctions were blurred between advocacy, health and social care support and research. Finally, we made efforts to reflect the differences in perspective from various stakeholders in the final phase as a public ranking to offer the opportunity to respond to this consultation process We cannot rule out a bias towards one or other respondent group as we did not proportionally weigh responses. On a special note, the priority of palliative care at home was integrated as part of phase III community-based services, not as a stand-alone research priority need. The process of research priority setting was complex due to multiple engagement phases, however the use of participatory approach underpinned by PPI principles enabled us to identify, address and integrate the diverse stakeholders values and perspectives ^33^ throughout the process.

Despite these caveats, the research prioritisation exercise was the first multi-stakeholder participatory approach focused on a broadened scope of rare diseases research in Ireland. There is a need for an ongoing engagement with the RAinDRoP expert group to establish plans for translation of the research priorities into actual research via policies and funding ^34^. Also, to create more patient and public awareness about European wide rare diseases research potential where patients and their families could be part of the research process, for example, ERN, EJP etc. ^12, 31^. Findings from the prioritisation exercise will inform future collaborative research programmes, networking opportunities, joint grants and research engagement events.

Since there is a significant variation in the use of research priority-setting methodology worldwide, there is no gold standard or best practice for evaluating the process of research priority setting ^35^. For this research priority setting exercise we didn’t follow any checklist as part of the project design and engagement process; however, we came across REPRISE checklist and guidelines ^33^ and tried to refer this guide to facilitate comprehensive and transparent reporting of health research priority-setting exercises. 

### Implications for policy

Public support of research lends authenticity to research advocacy that it would otherwise be impossible to achieve. The combined public/academic/clinician approach to strategy is more relevant and compelling. Collaborative tools and partnership allowed ethical data sharing for and with patients, and along with co-designing interventions, this will aim at improving patient-reported outcomes ^36^. This activity did not focus on a specific disease but the shared challenges of rare disease. Through the inclusion of interdisciplinary researchers, clinicians and stakeholders, this workshop facilitated and fostered knowledge exchange between those working towards an improved quality of life for people living with a rare disease. Finally, this enabled setting up research priorities based on patients living with rare diseases (rather than their diagnosis specific), which can eventually feed into the emerging policy framework relating to the research session in the Irish Rare Disease National Plan ^2^, and rare disease plans and strategies in European member states and the World Health Organization.

### Implications for practice

The RAinDRoP research prioritisation activity ensures transforming Irish health and educational systems to increase rare diseases awareness. This type of engagement utilising the PPI approach builds trust between research institutions and society. Involving patients and public in the RAinDRoP project has been demonstrated that their involvement in the research process helped us to identity paucity of evidence currently available to address the experience of living with a rare disease. This form of funding supports engagement to strengthen partnership with HRB and other key stakeholders within the rare disease community, academia, patient, clinicians and public, and also increases responsiveness to societal needs through patient and public engagement.

## Conclusion

The results of the RAinDRoP research prioritisation reflected the key points from the initial 2012 consultation process on rare disease research as part of the national plan for rare diseases in 2014 ^2^. The National Plan on Rare Diseases for Ireland identified several research challenges ^2^, such as the lack of dedicated national funding for rare disease research in Ireland. If this situation does not change, it will be a significant challenge for the rare disease community to translate research priorities into funded research projects. Conversely, the strengths of the RAinDRoP prioritisation include transparency and the high level of participation, engagement, involvement and agreement from a collective focus to inform future research to improve the experience and outcomes of people living with rare diseases in Ireland. It is the first do so research prioritisation exercise from a rare disease across life- span perspective. Hence, we encourage researchers, funding bodies and other stakeholders to use these priority statements as guidelines for future research work on rare diseases to maximise patient voice via patient and public involvement in research.

### Ethics approval and consent to participate

All participants received a comprehensive information sheet that outlined the nature and purpose of each survey, along with issues related to consent, confidentiality, voluntary participation and the rights of withdrawal from the survey.

We obtained an exemption from the full ethics review by the University College Dublin Research Ethics Committee (LS-E-19-32-Somanadhan).

## Data availability

### Underlying data

Figshare: RAinDRoP Data Set, https://doi.org/10.6084/m9.figshare.11984424.v5 ^22^.

This project contains the following underlying data (available in one PDF document):

- File 1: Statements received from the phase I surveys
- File 2: Initial grouping of statements (n=1015) into questions from the phase I surveys
- File 3: All café priority-based data on high importance and investment
- File 4: Priority ratings in terms of ‘importance’ by café group in phase II
- File 5: Priorities ranked in the first position by respondents in FWPCPS

### Extended data

Figshare: RAinDRoP Data Set, https://doi.org/10.6084/m9.figshare.11984424.v5 ^22^.

This project contains the following extended data (available in one PDF document):

- File 1: PCSRRDI Participant Information Sheet
- File 2: Phase I survey
- File 3: RPW Workshop agenda
- File 4: Follow-up Public Consultation and Prioritisation Survey (FWPCPS)
- File 5: RAinDRoP Project Timelines

Data available under the terms of the Creative Commons Attribution 4.0 International license (CC-BY 4.0).
