# Supplementary material for: Rare Disease Research Partnership (RAinDRoP): a collaborative approach to identify research priorities for rare diseases in Ireland
Source: HRB Open Res. 2020 Nov 11;3:13. Originally published 2020 Apr 14. [Version 2] doi: 10.12688/hrbopenres.13017.2 (PMC7702160; doi:10.12688/hrbopenres.13017.2)
Supplement: Peer review history

# Peer review: Reviewer response for version 2

**DOI:** 10.21956/hrbopenres.14298.r28318

**Published:** 2020-11-27

**Authors:** Anneliese Synnot

**Affiliations:** 1 Centre for Health Communication and Participation, School of Psychology and Public Health, La Trobe University, Melbourne, Australia

The manuscript is vastly improved. Well done.

Is the work clearly and accurately presented and does it cite the current literature?

Partly

If applicable, is the statistical analysis and its interpretation appropriate?

Not applicable

Are all the source data underlying the results available to ensure full reproducibility?

Yes

Is the study design appropriate and is the work technically sound?

Yes

Are the conclusions drawn adequately supported by the results?

No

Are sufficient details of methods and analysis provided to allow replication by others?

Partly

Reviewer Expertise:

Research priority setting, consumer involvement in research.

I confirm that I have read this submission and believe that I have an appropriate level of expertise to confirm that it is of an acceptable scientific standard.

# Peer review: Reviewer response for version 2

**DOI:** 10.21956/hrbopenres.14298.r28319

**Published:** 2020-11-25

**Authors:** Helen McAneney, Julie McMullan

**Affiliations:** 1 Queen's University Belfast, Belfast, UK; 2 Northern Ireland Rare Disease Partnership, Newtownards, UK

We wish to thank the authors for taking our suggestions on board and for the revisions they have made in light of these comments. The article now reads more clearly and succinctly throughout. We are therefore happy for the article to be indexed in its current form. These research priorities are timely and will assist in the future direction of rare disease research in Ireland. We wish the research team well in their continued efforts and work of the RAinDRop consortium.

Is the work clearly and accurately presented and does it cite the current literature?

Partly

If applicable, is the statistical analysis and its interpretation appropriate?

Not applicable

Are all the source data underlying the results available to ensure full reproducibility?

Yes

Is the study design appropriate and is the work technically sound?

Yes

Are the conclusions drawn adequately supported by the results?

Partly

Are sufficient details of methods and analysis provided to allow replication by others?

Partly

Reviewer Expertise:

Rare diseases, methodology, qualitative studies.

We confirm that we have read this submission and believe that we have an appropriate level of expertise to confirm that it is of an acceptable scientific standard.

# Peer review: Reviewer response for version 1

**DOI:** 10.21956/hrbopenres.14110.r27709

**Published:** 2020-08-17

**Authors:** Helen McAneney, Julie McMullan

**Affiliations:** 1 Northern Ireland Rare Disease Partnership, Newtownards, UK; 2 Queen's University Belfast, Belfast, UK

**Overall Comments**

 This study is timely and very much needed. It has been conducted to a high level and with much thought given to the various voices, as evident from the level of PPI throughout. The authors have endeavored to obtain the views and opinions of all stakeholders, and to reduce bias through the multi-phase approach. However, the manuscript does not convey the study as clearly as it might. The methods and results section require clearer articulation, particularly as there are elements of methods in the result section and vice versa. A flow diagram through the various phases and how one informed the next would be beneficial. Lastly, do make more of the findings, rather than the process, as the 15 priorities are the central aspect of the study’s aims and objectives.

**Title**

 I would suggest adding ‘Ireland’ to the title if the priorities are specifically for this country and have been identified for this region only. 

**Abstract**

 The abstract requires more of a focus on the current study. The information given is confusing as the RAinDRoP project is overlapping and getting mixed in with the current study. Confusing for the reader to determine which information is relevant. 

 It is not clear in the abstract what the main conclusions are from this study, these should be stated clearly to enable the reader to gain an understanding of what the study has found and what implications the findings might have in a wider context. 

 Also suggest putting the keywords into alphabetical order.

 Specific comments/queries

1. Please include within the methods the timeframe of the entire study, or the phases.
2. Within the methods section the sentence “a shortlisting step by step ...” sentence is confusing. Please clarify this and explain what a ‘total score of above 50%’ means, as the ‘total score’ has not been explained here. You also mention 10-12 RQ per theme, but in the abstract themes are not explained – i.e. in the main text you have that there were 6 themes in phase I, yet in the abstract you only mention the 3 themes of phase II.
3. The steps of phase II are not clear, how does phase I results become those in phase III?
4. Conclusions – you mention that priorities were identified, but no where in the abstract are these given or summarised.

**Introduction**

 Within the first paragraph there is repetition of some facts, such as the individual rare yet collectively common, as well as the mentioning of 7,000 rare conditions/7,000 rare diseases.

 In the second paragraph, you mention the 1.4 billion Euros invested, but what is the time frame of this, is it recent, or over a period of time?

 It may be useful to mention that in rare disease research, that the patients are often the experts, due to the nature of the conditions, and so the greater importance of including the patient and carer’s voice in the priority setting exercise, rather than just academics and HCPs.

**Aims and objectives**

 The aims and objectives of the study need a clearer focus on the current study. Again, confusion between this study and RAinDRoP make it difficult for the reader to determine the relevance for the current project. Would suggest moving the overall RAinDRoP aims and objectives into the background section and only give the aims and objectives of the prioritization exercise in the aims and objectives section.

**Methods**

 In the methods section a rationale should be provided for the methodology chosen. Was consideration given to other methods? What were the benefits or potential pitfalls of the method chosen? For instance, why were three phases conducted and not just two?

 Although Table 1 is useful, it would be preferred that this is developed into a flow diagram that then leads the reader through the three phases, what is obtained in each and how progress to the next phase occurs. This would summarize the process and progress through the methods section and map the text to the figure.

 Phase I, you mention expert groups carried out the scoring of the statements – previously you mentioned just one expert group, so please explain who are ‘each expert group’.

 Phase II developed the top 15 research priorities, and then phase III took these and ranked them again – what does this ranking add in phase III, over and above the results of Phase II, as they are the same? What was the methodological thinking being the third phase?

 How were the 3 themes chosen for the Phase 3 workshop? 

 How did the 29 themes identified in Phase 1 become 3 themes for discussion in Phase 2? More detail is needed as to how this process happened.

 The abbreviations for the three phases are difficult to read, and not helpful. Please think of rephrasing them.

 PPI has been central to this work from the beginning which is very encouraging to read. 

 Some information included in the ‘Methods’ section that should be moved to the ‘Discussion’ e.g. *The workshop created an opportunity for information-sharing and an open dialogue around the challenges faced by a rare disease, as well as its future direction. Relationships built between researchers and those with lived experience have the potential to extend to future collaborations.*

 Also some information in the ‘methods’ is actually ‘results’ e.g., “ *From this ranking 29 themes or statements identified from the surveys were brought forward for discussion at the phase II workshop”.*

**Results**

 Similarly, the results section includes, ‘ *Each participant was given sticker sets and play money (as described in the Methods). Participants were then asked to assign a level of importance and feasibility to each subtheme using the colour coded stickers. To further clarify their decision-making, each participant was asked to invest the play money as they saw fit – to put their money where their mouth is! Data was sorted by % of high priorities, and then % of euro investment. Applied heat mapping to show which themes are more dominant within the high priority group are available as Underlying data: File3.’ *This should be in the ‘Methods’ section. I suggest rereading the ‘Methods’ and ‘Results’ to ensure the information is included in the correct sections.

 There are abbreviations in this section, not given in full or listed under abbreviations, e.g. PwRDs, HCPs.

 Figure 2, there is a typo as the number of responses were 1,015, and not 10015.

 Table 5 and Table 6

- Table 5 are the 15 priorities identified in Phase 2. Table 6 and the same 15 priorities but re-ranked. If so, then where does the ‘Palliative care at home’ in Table 5 go as it is not in Table 6, and likewise in Table 6 we have ‘Education and Training’ which is not in Table 5.
- Table 5, it would be worth a foot note to say that the percentages add up to approx. 72% as these are the statements with the highest %.
- Table 5, you have a ‘psychosocial impact’ and a ‘psychosocial impact of a diagnosis’ listed, please clarify how these are different.
- Table 6, this is the re-ranking of the 15 priorities, but it would be useful to show the change in positioning, e.g. 2 ^nd^ in Table 5 is now in 12 ^th^ place in Table 6. Is the ranking important and if so, does the change in position matter, was it expected, why the change etc. These points should be discussed in the discussion.

**Discussion**

 This is the section that should take the findings and summarise and expand on them. Are the listed 15 priorities what you expected to find? Are they the same as in other countries, or are some specific to Ireland? What did the re-ranking do/add to the study? Please reflect on your findings and discuss.

**Conclusion**

 This section needs to be linked to the results more closely. Currently this section does not clearly display the main conclusions from the study. These should be made explicit in this section for the reader.

Is the work clearly and accurately presented and does it cite the current literature?

Partly

If applicable, is the statistical analysis and its interpretation appropriate?

Not applicable

Are all the source data underlying the results available to ensure full reproducibility?

Yes

Is the study design appropriate and is the work technically sound?

Yes

Are the conclusions drawn adequately supported by the results?

Partly

Are sufficient details of methods and analysis provided to allow replication by others?

Partly

Reviewer Expertise:

Rare diseases, methodology, qualitative studies.

We confirm that we have read this submission and believe that we have an appropriate level of expertise to confirm that it is of an acceptable scientific standard, however we have significant reservations, as outlined above.

# Response

**Published:** 2020-10-21

**Authors:** Suja Somanadhan

**Affiliations:** University College Dublin, Ireland

**Many thanks for your comments. We considered each in turn and, have amended the manuscript in light of these considerations. Other comments and questions we answer here.**

Overall Comments

This study is timely and very much needed. It has been conducted to a high level and with much thought given to the various voices, as evident from the level of PPI throughout. The authors have endeavoured to obtain the views and opinions of all stakeholders and to reduce bias through the multi-phase approach. However, the manuscript does not convey the study as clearly as it might. The methods and results section require clearer articulation, particularly as there are elements of methods in the result section and vice versa. A flow diagram through the various phases and how one informed the next would be beneficial. Lastly, do make more of the findings, rather than the process, as the 15 priorities are the central aspect of the study’s aims and objectives.

**The manuscript has been amended to address these recommendations.**

Title

I would suggest adding ‘Ireland’ to the title if the priorities are specifically for this country and have been identified for this region only.

**Tile has been amended as follows:** **Rare Disease Research Partnership (RAinDRoP): a collaborative approach to identify research priorities for rare diseases in Ireland**

Abstract

The abstract requires more of a focus on the current study. The information given is confusing as the RAinDRoP project is overlapping and getting mixed in with the current study. Confusing for the reader to determine which information is relevant.

It is not clear in the abstract what the main conclusions are from this study, these should be stated clearly to enable the reader to gain an understanding of what the study has found and what implications the findings might have in a wider context.

Specific comments/queries

1.         Please include within the methods the timeframe of the entire study, or the phases. **Amended**

2.         Within the methods section the sentence “a shortlisting step by step ...” sentence is confusing. Please clarify this and explain what a ‘total score of above 50%’ means, as the ‘total score’ has not been explained here. You also mention 10-12 RQ per theme, but in the abstract themes are not explained – i.e. in the main text you have that there were 6 themes in phase I, yet in the abstract you only mention the 3 themes of phase II. **Amended**

 3.        The steps of phase II are not clear, how does phase I results become those in phase III? **Amended**

4.         Conclusions – you mention that priorities were identified, but no where in the abstract are these given or summarised. **Amended**

**Response: Abstract restructured within the 300-word limit in light of three reviewer’s recommendations:**

**Background:**

**Rare diseases are individually rare, but collectively these conditions are common. Research on rare diseases are currently focused on disease-specific needs rather than a life-course perspective. The Rare Disease Research Partnership (RAinDRoP) was established in 2018 to bring together a wide variety of diverse voices in the rare disease community in Ireland and form a research partnership.**

**Methods: A participatory multiple phase approach was used to identify research priorities for rare diseases. The research process involved three main phases: Phase I, Public Consultation Survey(PCS); Phase II, Research Prioritisation Workshop (RPW); Phase III, Public Prioritisation Ranking Survey (PRS). The time frame for the entire study was from November 2018 to June 2019.**

**Results: In total, 240 individuals completed the phase I, of which only 96 survey participants provided information on their background,  32% (n=31) self-identified as a person living with a rare disease(s). One thousand and fifteen statements were collected, which reflected issues and shared challenges in rare diseases. MSExcel was used to gain frequencies and percentages.**

**Phase II was focused on three main themes (1) Route to Diagnosis (2) Living with Rare Disease (3) Integrated and Palliative Care. 42 participants engaged at each workshop. Seventy-five individuals completed the phase III prioritisation ranking survey and ranked the top 15 research priorities.  The top five priorities were (1)Support at the time of diagnosis, (2) Diagnostic test for rare diseases (3)Education and training (4) Patient voice (5) Data sharing and integration of services for rare diseases.**

**Conclusions: The research priorities identified here for rare diseases were developed jointly in collaboration with patients, families, healthcare professionals and policymakers. So, we encourage researchers, funding bodies and other stakeholders to use this priority list as a guiding document for future research work to improve the health and lives of people living with rare diseases.**

Also suggest putting the keywords into alphabetical order. **Amended**

Introduction

Within the first paragraph there is repetition of some facts, such as the individual rare yet collectively common, as well as the mentioning of 7,000 rare conditions/7,000 rare diseases.

**The manuscript has been amended  in light of this consideration**

In the second paragraph, you mention the 1.4 billion Euros invested, but what is the time frame of this, is it recent, or over a period of time?

**The manuscript has been amended  in light of this consideration**.

It may be useful to mention that in rare disease research, that the patients are often the experts, due to the nature of the conditions, and so the greater importance of including the patient and carer’s voice in the priority setting exercise, rather than just academics and HCPs.

**The manuscript has been amended  in light of this consideration**

Aims and objectives

The aims and objectives of the study need a clearer focus on the current study. Again, confusion between this study and RAinDRoP make it difficult for the reader to determine the relevance for the current project. Would suggest moving the overall RAinDRoP aims and objectives into the background section and only give the aims and objectives of the prioritization exercise in the aims and objectives section.

**Agreed and amended**

Methods

In the methods section a rationale should be provided for the methodology chosen. Was consideration given to other methods? What were the benefits or potential pitfalls of the method chosen? For instance, why were three phases conducted and not just two? Amended

**Having considered the various methodologies and schools of thoughts, participatory multiple method was chosen as a suitable methodological approach for this project. We felt this approach would be the most appropriate to reflect and promote participation from the patients and public involvement (PPI) perspective to focus and identify research priorities that address uncertainties of living with rare diseases. Participation in this study means that individuals are involved in Rare Disease research Partnership (RAinDRoP) was engaged in a meaningful way from the beginning of the process with a focus to improve the quality of the patient-focused rare diseases research and its impact.** **An expert group was formed to oversee this research prioritisation exercise and this group composed of members of the rare disease taskforce, patient organisation representatives (n=3); patients and families living with rare diseases (n=3); members of the National Rare Disease Office in Ireland (n=2), academics (n=2), researchers (n=2), healthcare professionals (n=2).**

Although Table 1 is useful, it would be preferred that this is developed into a flow diagram that then leads the reader through the three phases, what is obtained in each and how progress to the next phase occurs. This would summarize the process and progress through the methods section and map the text to the figure.

**Amended: Table is restructured into Figure 1  flow diagram that then leads the reader through the three phases.**

Phase I, you mention expert groups carried out the scoring of the statements – previously you mentioned just one expert group, so please explain who are ‘each expert group’.

**Participation in this study means that individuals are involved in Rare Disease research Partnership (RAinDRoP) was engaged in a meaningful way from the beginning of the process with a focus to improve the quality of the patient-focused rare diseases research and its impact. An expert group was formed to oversee this research prioritisation exercise and this group composed of members of the rare disease taskforce, patient organisation representatives (n=3); patients and families living with rare diseases (n=3); members of the National Rare Disease Office in Ireland (n=2), academics (n=2), researchers (n=2), healthcare professionals (n=2).**

Phase II developed the top 15 research priorities, and then phase III took these and ranked them again – what does this ranking add in phase III, over and above the results of Phase II, as they are the same? What was the methodological thinking being the third phase?

**In the prioritisation process, the focus on research may have been lost for some participants. This became evident in some of the survey responses and at the workshop discussions where distinctions were blurred between advocacy, health and social care support and research. Finally, we made efforts to reflect the differences in perspective from various stakeholders in the final phase as a public ranking to offer the opportunity to respond to this consultation process.**

How were the 3 themes chosen for the Phase 3 workshop?

**Research Prioritisation Workshop was Phase II**

How did the 29 themes identified in Phase 1 become 3 themes for discussion in Phase 2? More detail is needed as to how this process happened.

**The three emerging themes identified via online survey phase I became the focus of the Research Prioritisation Workshop (RPW) Phase II.**

The abbreviations for the three phases are difficult to read, and not helpful. Please think of rephrasing them.

**The manuscript has been amended in light of these recommendations.**

**Phase I, Public Consultation Survey(PCS); Phase II, Research Prioritisation Workshop (RPW); Phase III, Public Prioritisation Ranking Survey (PRS).**

Some information included in the ‘Methods’ section that should be moved to the ‘Discussion’ e.g. The workshop created an opportunity for information-sharing and an open dialogue around the challenges faced by a rare disease, as well as its future direction. Relationships built between researchers and those with lived experience have the potential to extend to future collaborations.

**The manuscript has been amended in light of these recommendations.**

Also some information in the ‘methods’ is actually ‘results’ e.g., “From this ranking 29 themes or statements identified from the surveys were brought forward for discussion at the phase II workshop”.

**The manuscript has been amended in light of these recommendations**.

Results

Similarly, the results section includes, ‘Each participant was given sticker sets and play money (as described in the Methods). Participants were then asked to assign a level of importance and feasibility to each subtheme using the colour coded stickers. To further clarify their decision-making, each participant was asked to invest the play money as they saw fit – to put their money where their mouth is! Data was sorted by % of high priorities, and then % of euro investment. Applied heat mapping to show which themes are more dominant within the high priority group are available as Underlying data: File3.’ This should be in the ‘Methods’ section. I suggest rereading the ‘Methods’ and ‘Results’ to ensure the information is included in the correct sections. 

**The manuscript has been amended in light of these recommendations**.

There are abbreviations in this section, not given in full or listed under abbreviations, e.g. PwRDs, HCPs.

**The manuscript has been amended in light of these recommendations,  see below**

**People living with a rare disease(PwRDs) Health Care Practitioners (HCPs)**

Figure 2, there is a typo as the number of responses were 1,015, and not 10015. **Corrected**

Table 5 and Table 6

•           Table 5 are the 15 priorities identified in Phase 2. Table 6 and the same 15 priorities but re-ranked. If so, then where does the ‘Palliative care at home’ in Table 5 go as it is not in Table 6, and likewise in Table 6 we have ‘Education and Training’ which is not in Table 5.

**All topics from the research prioritisation workshop (RPW) Phase II were included, and respondents were asked to rank these areas in order of importance.  From this, a final set of research priorities were identified to inform the phase III Public Prioritisation Ranking Survey (PRS).**

**Community-based services, treatment, multi-morbidity was discussed in terms of delivering care closer to home in an integrated way. This category included palliative care needs for individuals living with rare progressive and complex illness. Education and training were also one of the priority identified form RPW.  See above answer  and the manuscript has been amended in light of these recommendations**

•           Table 5, it would be worth a foot note to say that the percentages add up to approx. 72% as these are the statements with the highest %.

**Amended**

•           Table 5, you have a ‘psychosocial impact’ and a ‘psychosocial impact of a diagnosis’ listed, please clarify how these are different.

**Psychosocial impact of living with rare diseases, this encompasses the effects on education and employment opportunities, stigmatisation, friendships etc. and the second priority was focused on the psychosocial impact of rare disease diagnosis**.

•           Table 6, this is the re-ranking of the 15 priorities, but it would be useful to show the change in positioning, e.g. 2nd in Table 5 is now in 12th place in Table 6. Is the ranking important and if so, does the change in position matter, was it expected, why the change etc. These points should be discussed in the discussion.

**This priority exercise was a co-designed at every stage of the process from the concept design, survey design, thematic analysis, workshop and the final ranking. The exercise aimed to maximise the impact of the rare disease community in Ireland. Therefore, the public ranking survey was important to offer an equal opportunity to respond to this consultation process. Hence the change in position of priority list doesn’t affect the overall process**.

Discussion

This is the section that should take the findings and summarise and expand on them. Are the listed 15 priorities what you expected to find? Are they the same as in other countries, or are some specific to Ireland? What did the re-ranking do/add to the study? Please reflect on your findings and discuss.

**The manuscript has been amended in light of these recommendations**

**Conclusion**

This section needs to be linked to the results more closely. Currently this section does not clearly display the main conclusions from the study. These should be made explicit in this section for the reader.

**The manuscript has been amended in light of these recommendations**

# Peer review: Reviewer response for version 1

**DOI:** 10.21956/hrbopenres.14110.r27415

**Published:** 2020-05-29

**Authors:** Anneliese Synnot

**Affiliations:** 1 Centre for Health Communication and Participation, School of Psychology and Public Health, La Trobe University, Melbourne, Australia

**Overall comments**

1. The project seems well justified and conducted, with plenty of consumer/patient input. I got a little lost at some points in the methods and results. In some cases there needs to be more detail, or in others I would like to see a little more justification of some of the methods decisions made. Then in the discussion, there could be more consideration of how these decisions might’ve affected the results
2. The authors might also consider re-structuring the methods and results. It seems a little muddled in places as to what goes in the methods and what goes in the results. Suggest results should stick to participant numbers and demographics and research priorities generated/prioritised.
3. The flow of priorities through the project (particularly the numbers) were unclear to me. Would appreciate a flow diagram.
4. It's hard to get a sense of your main results as these are not summarised in the abstract, nor the discussion or conclusion. Instead, you focus on the process you used to generate the results. The reader is left to pick apart quite a number of different tables and results presented in the results section. A concise summary would help.
5. Consider using the REPRISE reporting checklist for research priority setting exercises (Tong *et al. *(2019) ^1^) to ensure completeness of reporting. 

**Abstract**

 In the results section:

1. Suggest adding how many of the 240 participants were patients and carers.
2. This part seems a little muddled and some of what you have here seems more appropriate for the methods (i.e. how the process worked).
3. It also seems important to describe what the final 15 research priorities actually were (or some kind of summary) in the results, so I suggest that should be your focus here.
4. It seems like there is a line missing between the second and third sentences. You go from mentioning 1000-odd suggestions to those that scored more than 50%. It would be helpful to have the process explained here (but perhaps in the methods).
5. The words, “A shortlisting step by step” don’t make sense. Is there a word missing?
6. How did you go from 10-12 statements at the workshop to 15 in the final survey?

**Aims and objectives**

1. These seem a little muddled between the aims of RAINDROP and the aims of the priority setting exercise. Given you state in the background, “This article reports on a rare disease research prioritization exercise” consider tightening the focus onto the aims of the prioritisation exercise (i.e. to identify rare disease research priorities for Ireland from multiple stakeholder perspectives). The aims and objectives of RAINDROP could go in the background.

**Methods**

1. Please add a line stating what type of research you were wanting to prioritise (i.e. interventions, psychosocial etc).
2. Please provide more detail about the process you used (literature and policy document review then I assume some form of thematic analysis) to generate the six key topics about which respondents were asked.
3. It would be helpful if you could justify why you framed the questions around the six key topics, instead of leaving the survey open-ended (i.e. just asking what questions people wanted answered in research) and then grouping the responses into inductive themes. Your *a priori* grouping of the questions would’ve shaped the final priorities generated. Perhaps this could also be discussed in the discussion.
4. I notice you advertised the exercise (in the flyer in your supplementary material) as being about finding top 10 priorities, but you ended up with 15. It would be good to be explicit about this in the methods and why you increased it to 15.
5. It would be helpful if you could create a flow diagram to show to number of priorities/themes generated at each stage of the project. It's currently a little confusing.
6. Can you explain why you asked people to rank importance, feasibility and assign euros when it seems that you only considered their score for importance when generating the top priorities?
7. Can you explain why, when you generated a prioritised list of 15 research topics, you then went back out to public consultation to seek further input into this prioritisation? I’m not sure of the value when the 15 priorities remained the same, but their order might’ve changed a little.

**Results**

1. In the section, ‘PhaseII: RPW’ you have a lot of information that is more appropriate in the methods section (some of it is repeated there). Suggest you stick to describing the participants and the results of the prioritisation here.
2. I’m a bit confused about the flow of priorities in the workshop. So you had 29 sub-themes to start with, and then you took the top 3 from each of the three overall themes to get to a top 10 (with one theme having two equal highest scoring themes), but how did you get to a top 15? And why do you report a top 10 and a top 15?
3. In the section, Phase III: FWPCPS, suggest you describe at least the top five priorities in the text.
4. Based on your supplementary material it looks like you took research priorities framed as questions into the workshop (grouped under different areas) but you came away with priorities framed as thematic areas. It's not clear why this happened. Assumedly, the priorities framed as questions would be very helpful to researchers. The statements you have accompanying the top 15 priorities are helpful but could still be framed as a myriad of different research questions. I wonder if you lost something valuable in this transition?

**Discussion**

1. Suggest you provide a summary of the main findings at the start of the discussion. It may be helpful to ‘unpack’ some of your secondary findings in terms of differences between stakeholder groups, shifting of relative priorities between workshop and final survey and how results compared between importance/feasibility and funding rankings.
2. As mentioned earlier, a deeper reflection on how some of your methods decisions may have affected the results would be helpful.

**Minor points**

1. The acronyms used for each of the three phases are not helpful. Consider using the terms in full or shortening to ‘Initial survey’, ‘workshop’ and ‘final survey’ or similar.

Is the work clearly and accurately presented and does it cite the current literature?

Partly

If applicable, is the statistical analysis and its interpretation appropriate?

Not applicable

Are all the source data underlying the results available to ensure full reproducibility?

Yes

Is the study design appropriate and is the work technically sound?

Yes

Are the conclusions drawn adequately supported by the results?

No

Are sufficient details of methods and analysis provided to allow replication by others?

Partly

Reviewer Expertise:

Research priority setting, consumer involvement in research.

I confirm that I have read this submission and believe that I have an appropriate level of expertise to confirm that it is of an acceptable scientific standard, however I have significant reservations, as outlined above.

## References

[1] : Reporting guideline for priority setting of health research (REPRISE). *BMC Medical Research Methodology*.2019;19(1) : 10.1186/s12874-019-0889-3 10.1186/s12874-019-0889-3 31883517PMC6935471

# Response

**Published:** 2020-10-21

**Authors:** Suja Somanadhan

**Affiliations:** University College Dublin, Ireland

**Many thanks for your comments. We considered each in turn and, have amended the manuscript in light of these considerations. Other comments and questions we answer here**

- The project seems well justified and conducted, with plenty of consumer/patient input. I got a little lost at some points in the methods and results. In some cases, there needs to be more detail, or in others I would like to see a little more justification of some of the methods decisions made. Then in the discussion, there could be more consideration of how these decisions might’ve affected the results.

**The methods and discussion sessions have been amended to address these recommendations.**

- The authors might also consider re-structuring the methods and results. It seems a little muddled in places as to what goes in the methods and what goes in the results. Suggest results should stick to participant numbers and demographics and research priorities generated/prioritised.

**The method and results sessions have been amended to address these recommendations.**

- The flow of priorities through the project (particularly the numbers) were unclear to me. Would appreciate a flow diagram.

 
**Figure I has been amended to illustrate the project phases (see Figure 1)**

- It's hard to get a sense of your main results as these are not summarised in the abstract, nor the discussion or conclusion. Instead, you focus on the process you used to generate the results. The reader is left to pick apart quite a number of different tables and results presented in the results section. A concise summary would help.

**The abstract has been restructured** **within the 300-word limit in light of three reviewer’s recommendations** **to provide a concise summary.**

**Background:**

**Rare diseases are individually rare, but collectively these conditions are common. Research on rare diseases are currently focused on disease-specific needs rather than a life-course perspective. The Rare Disease Research Partnership (RAinDRoP) was established in 2018 to bring together a wide variety of diverse voices in the rare disease community in Ireland and form a research partnership.**

**Methods: Participatory multiple phases approach were used to identify research priorities for rare diseases. The research process involved three main phases: Phase I, Public Consultation Survey(PCS); Phase II, Research Prioritisation Workshop (RPW); Phase III, Public Prioritisation Ranking Survey (PRS). The time frame for the entire study was from November 2018 to June 2019.**

**Results: In total, 240 individuals completed the phase I, of which only 96 survey participants provided information on their background,  32% (n=31) self-identified as a person living with a rare disease(s). One thousand and fifteen statements were collected, which reflected issues and shared challenges in rare diseases. MSExcel was used to gain frequencies and percentages.**

**Phase II was focused on three main themes (1) Route to Diagnosis (2) Living with Rare Disease (3) Integrated and Palliative Care. Forty-two participants engaged at each workshop. Seventy-five individuals completed the phase III prioritisation ranking survey and ranked the top 15 research priorities.  The top five priorities were (1)Support at the time of diagnosis, (2) Diagnostic test for rare diseases (3)Education and training (4) Patient voice (5) Data sharing and integration of services for rare diseases.**

**Conclusions: The research priorities identified here for rare diseases were developed jointly in collaboration with patients, families, healthcare professionals and policymakers. So, we encourage researchers, funding bodies and other stakeholders to use this priority list as a guiding document for future research work to improve the health and lives of people living with rare diseases.**

Consider using the REPRISE reporting checklist for research priority setting exercises (Tong et al. (2019)1) to ensure completeness of reporting.

**Thank you for sharing this checklist. W** **e amended reporting  followed by REPRISE checklist and guidelines**

Abstract

In the results section:

- Suggest adding how many of the 240 participants were patients and carers.

**Amended **

- This part seems a little muddled and some of what you have here seems more appropriate for the methods (i.e. how the process worked).

**Amended**

- It also seems important to describe what the final 15 research priorities actually were (or some kind of summary) in the results, so I suggest that should be your focus here.   

**Amended**

- It seems like there is a line missing between the second and third sentences. You go from mentioning 1000-odd suggestions to those that scored more than 50%. It would be helpful to have the process explained here (but perhaps in the methods).

**Corrected **

- The words, “A shortlisting step by step” don’t make sense. Is there a word missing?

**Amended**

- ​How did you go from 10-12 statements at the workshop to 15 in the final survey?                

**Amended**

Abstract restructured within the 300-word limit in light of three reviewer’s recommendations (see above)

Aims and objectives

- These seem a little muddled between the aims of RAINDROP and the aims of the priority setting exercise. Given you state in the background, “This article reports on a rare disease research prioritization exercise” consider tightening the focus onto the aims of the prioritisation exercise (i.e. to identify rare disease research priorities for Ireland from multiple stakeholder perspectives). The aims and objectives of RAINDROP could go in the background.

**Amended**

Methods

- Please add a line stating what type of research you were wanting to prioritise (i.e. interventions, psychosocial etc).

**Amended **

- Please provide more detail about the process you used (literature and policy document review then I assume some form of thematic analysis) to generate the six key topics about which respondents were asked.

 
**The manuscript has been amended  in light of these considerations**

- It would be helpful if you could justify why you framed the questions around the six key topics, instead of leaving the survey open-ended (i.e. just asking what questions people wanted answered in research) and then grouping the responses into inductive themes. Your a priori grouping of the questions would’ve shaped the final priorities generated. Perhaps this could also be discussed in the discussion.

**The process of research priority setting for rare diseases can be complex due to heterogeneity, and each rare disease impacts a small population. However, collectively many individuals are affected by these conditions. Therefore, identifying research prioritisation on each rare illness can be challenging in a single priority setting to identify, address and integrate the different perspectives from estimated 7000 rare diseases.  Hence, the expert group collaboratively designed the initial framework of the survey from a life-course perspective rather than a disease-specific focus.**

 

- I notice you advertised the exercise (in the flyer in your supplementary material) as being about finding top 10 priorities, but you ended up with 15. It would be good to be explicit about this in the methods and why you increased it to 15.

**The manuscript has been amended  in light of these considerations**

- It would be helpful if you could create a flow diagram to show to number of priorities/themes generated at each stage of the project. It's currently a little confusing.

**Amended **

- Can you explain why you asked people to rank importance, feasibility and assign euros when it seems that you only considered their score for importance when generating the top priorities?

**Participants were then given colour-coded stickers to assign a level of importance, and feasibility to each subtheme. To further clarify their decision-making and encourage active participation, each participant was given play money and asked to invest it as they saw fit – to put their money where their mouth is! However, we only considered their score for importance when generating the top priorities due to indifference approach to feasibility across three workshops**.  

- Can you explain why, when you generated a prioritised list of 15 research topics, you then went back out to public consultation to seek further input into this prioritisation? I’m not sure of the value when the 15 priorities remained the same, but their order might’ve changed a little.

**In the prioritisation process, the focus on research may have been lost for some participants. This became evident in some of the survey responses or café discussions where distinctions were blurred between advocacy, health and social care support and research. Finally, we made efforts to reflect the differences in perspective from various stakeholders in the final phase as a public ranking to offer the opportunity to respond to this consultation process**.

Results

- In the section, ‘PhaseII: RPW’ you have a lot of information that is more appropriate in the methods section (some of it is repeated there). Suggest you stick to describing the participants and the results of the prioritisation here.

**Amended**

 

- I’m a bit confused about the flow of priorities in the workshop. So you had 29 sub-themes to start with, and then you took the top 3 from each of the three overall themes to get to a top 10 (with one theme having two equal highest scoring themes), but how did you get to a top 15? And why do you report a top 10 and a top 15?

**Amended**

- In the section, Phase III: FWPCPS, suggest you describe at least the top five priorities in the text.

** In light of all reviewer’s recommendations, it has been included as part of the results**.

- Based on your supplementary material it looks like you took research priorities framed as questions into the workshop (grouped under different areas) but you came away with priorities framed as thematic areas. It's not clear why this happened. Assumedly, the priorities framed as questions would be very helpful to researchers. The statements you have accompanying the top 15 priorities are helpful but could still be framed as a myriad of different research questions. I wonder if you lost something valuable in this transition?

**The rare disease research-related topic areas were developed from input solicited through a multiphase process such as public survey, research prioritisation workshop and prioritisation ranking survey.  The 15 rare disease research priorities were ranged very broadly from diagnosis, support at the time of diagnosis, to challenges of day to day life living with rare diseases, and integrated and palliative care. We felt these research priorities represent key strategic areas that are executional in nature. We agreed to keep priorities are categorized within broader themes to represent the view of the patient, family and healthcare professionals across life-span, rather than narrow it down to a research question specific priority with a focus on particular rare disease.**

Discussion

- Suggest you provide a summary of the main findings at the start of the discussion. It may be helpful to ‘unpack’ some of your secondary findings in terms of differences between stakeholder groups, shifting of relative priorities between the workshop and final survey and how results compared between importance/feasibility and funding rankings.

**The manuscript has been amended in light of these recommendations.**

- As mentioned earlier, a deeper reflection on how some of your methods decisions may have affected the results would be helpful.

**The manuscript has been amended in light of these recommendations**
